# The current scenario, challenges, and their possible mitigation practices for livestock production potentials in the Haor ecosystem of Bangladesh: A review

**DOI:** 10.5455/javar.2025.l998

**Published:** 2025-12-25

**Authors:** Shubash Chandra Das, Mohammad Mahbubul, Jannatul Ferdows, Mohini Paul

**Affiliations:** 1 Department of Poultry Science, Bangladesh Agricultural University, Mymensingh, Bangladesh; 2 Department of Animal Breeding and Genetics, Bangladesh Agricultural University, Mymensingh, Bangladesh

**Keywords:** Haor livestock, Haor ecosystem, Climate, Livelihood, Mitigation practices

## Abstract

This review paper provides comprehensive insights into the current livestock production potential in the Haor regions, the impact of climate change on livestock production, development strategies for the sector, challenges associated with climate-resilient livestock farming, and potential mitigation measures. The Haors of Bangladesh, situated in the greater Sylhet and Mymensingh districts, are vital components of the ecosystem, holding significant economic, ecological, and commercial importance. The unique features of these low-lying floodplains, including their resources and seasonal variations, foster diverse biodiversity and ecosystems, covering over 1.99 million hectares and home to approximately 19.37 million people. All authors independently gathered data from peer-reviewed articles, dissertations, theses, government agencies, and media reports using PubMed, Web of Science, and Google Scholar from January 2023 to June 2025. Data were also collected through personal communication and consultation with livestock officers from the Department of Livestock Services serving in the Haor districts and relevant experts (*n* = 30). Climatic data were obtained from the Climate Information Management System of the Bangladesh Agricultural Research Council. Residents in Haor areas, particularly women and the elderly, are heavily engaged in livestock husbandry as a means of livelihood. The livestock sector is expected to meet nutritional requirements, generate employment, and provide national benefits through increased production of milk, meat, and eggs. This review paper is the first to synthesize, analyze, and interpret insights on the existing livestock production potential in Haor areas, along with challenges and possible mitigation strategies, culminating in some recommendations at the end of this paper.

## Introduction

**Figure 1.**
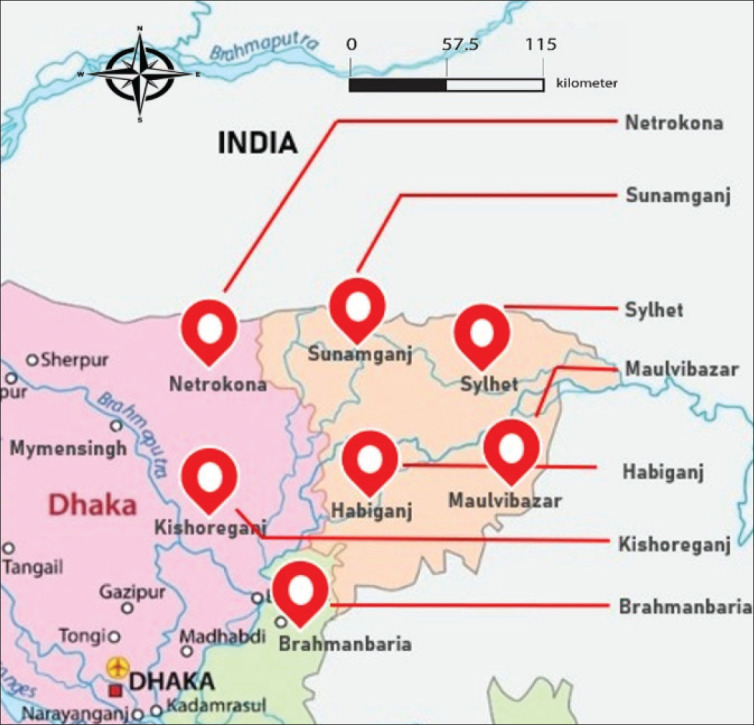
Map of Bangladesh. The location pins show the Haor region in the north-east areas.

Haors are back swamps or bowl-shaped depressions between the natural levees of a river, characterized by a unique wetland ecosystem and regular annual floods that cause them to remain underwater for over 6 months each year, starting with the monsoon [1–3]. Located in the northeastern part of the country, these areas are approximately 7–8 m below mean sea level and comprise large natural depressions, including the floodplains of the Meghna tributaries [4]. Figure 1 shows the Haor region of Bangladesh. It accounts for around 15% of the country's total land area and features various permanent and seasonal rivers, canals, “*beels*”, lakes, ponds, and extensive floodplains, all of which are abundant in valuable natural aquatic plants [1]. The Meghna basin is part of the larger Ganges-Brahmaputra-Meghna depression, which primarily includes core Haor areas in Sunamganj, Habiganj, Moulvibazar, and Sylhet districts but also extends into parts of Kishoreganj, Netrokona, and Brahmanbaria districts. There is a total of 373 Haors, with 95 in Sunamganj covering 268,531 hectares, 105 in Sylhet covering 189,909 hectares, 97 in Kishoreganj covering 133,943 hectares, 52 in Netrokona covering 79,345 hectares, 14 in Habiganj covering 109,514 hectares, 7 in Brahmanbaria covering 29,616 hectares, and 3 in Moulvibazar covering 47,602 hectares [5]. The total areas of these 7 districts are 19,99,800 hectares, of which the actual Haor areas span a total of 858,460 hectares [5]. The larger and more prominent Haors include Hakaluki*,* Saneer*,* Dekar*,* Tanguar*,* Gungiajuri*,* Maker*,* and Kawadighi [6]. The total population of these 7 Haor districts is approximately 19.37 million, which represents around 10% of Bangladesh's entire population [7]. The Haor region has considerable potential for increasing the productivity of agricultural food products, which can be achieved through suitable technology and management interventions [8]. The large water bodies in Haor regions are abundant in productive resources, encompassing flora and fauna, which should be investigated and utilized more effectively to augment crop, livestock, and fisheries production. Some livestock products, such as milk, meat, and eggs, can serve as unique sources of protein and energy, enhancing the livelihoods of Haor people. Establishing an effective market channel can enhance the linkage of various wetland ecosystems and underdeveloped Haor regions with the plain land market systems, including the capital city of Dhaka [9]. The Haor region of Bangladesh has been identified as one of the six hotspots under the Bangladesh Delta Plan (BDP)-2100, targeting strategies to enhance the livelihoods of the Haor people through climate-resilient agricultural production, improved water security, and sustainable development.

Haor regions exhibit three prominent seasons, of which the first is “peak inundation,” when all the reeds, grasses, and other aquatic plants are completely submerged and remain underwater for about 5 months. An acute problem arose during this season with cattle, goats, sheep, and other large animals due to widespread and prolonged flooding. This leads to a severe shortage of feed and fodder, more frequent diseases, and challenges with housing and shelter for animals and birds. After this, the water gradually recedes during the post-monsoon period, revealing various microhabitats, including swamps, reedlands, grasslands, mudflats, canals, natural levees, and rivers. These microhabitats gradually develop distinct shapes, exhibiting unique morphological features that are beneficial for livestock production. The winter season, a unique ecological feature of the Haor, lasts from November to February and is also the most crucial period for cultivating “*boro*” rice, a seasonal, short-duration paddy that serves as the main food source for the Haor community. During winter, the entire Haor basin is filled with newly sprouted green grasses and forages, providing the primary nutrition for the large number of animals kept there. However, the ducks and other migratory birds face challenges during winter due to the scarcity of natural food sources, such as snails, aquatic plants, insects, and small fish, especially at the end of winter, when the Haor basin becomes completely dry. Although floods and flash floods during the monsoon season have long been a regular phenomenon in this area, the situation has further deteriorated in recent years, with unpredictable natural disasters. Early flash floods have emerged in an episodic manner over the last decade, typically occurring just before the “*boro*” crops are harvested, causing significant economic damage to farmers. Livestock, fisheries, and other crops are also impacted directly or indirectly by these unpredictable natural incidents. The early monsoon “flash floods”, for example, struck the Haor region of Bangladesh in 2017, inflicting extensive damage to livestock and leading to considerable post-flood household insecurity due to the loss of regular income for impoverished farmers who relied on livestock and its products [10].

The causes of such natural calamities remain unclear; however, the unique Haor environment is likely at risk of deterioration due to climate change, hill cuts and deforestation, excess rainfall in the upstream hilly areas, river sedimentation, unplanned structural development, and destruction of native flora and fauna. These factors may contribute to rising temperatures and precipitation, unpredictable monsoon waves, increased flood intensity, and other natural disasters. Further, rainfall patterns and intensity have shifted noticeably, with more rain falling during the pre-monsoon season (overall climatic dynamics and their impact on the Haor ecosystem), which often coincides with harvest time and affects the safe harvest of “*boro*” rice [8]. This may also negatively impact livestock and fisheries production. Dey et al. [3] had been foreseeing the possibility of increasing the frequency and intensity of flash floods in the upcoming years. All these together result in a distinct and challenging lifestyle for the Haor residents [11], along with significant hardships for livestock and poultry [12].

Notwithstanding the plentiful natural resources, insufficient attention has been paid to exploring the potential for utilizing these resources to enhance the livelihoods of Haor communities. Neither the government nor any other authority has proposed a specific, comprehensive, and long-term project aimed at agricultural growth, particularly in livestock, to boost the economic prosperity of this region. Despite the threats that climate change poses to crops, livestock, and millions of people, there are few mitigation plans and requisite project activities from the pertinent authorities that may significantly safeguard them from natural disasters. Taking all these issues into account, this review paper focuses on the current state of natural resources in the Haor region, the livestock production potential, the impact of climate change and associated challenges, and finally recommends necessary mitigation strategies.

## Materials and Methods

### Literature search strategy

Each author individually contributed to the comprehensive search and data collection process to ensure sufficient coverage of the relevant literature reviews. The search mainly focused on including peer-reviewed journals, government reports, and credible media sources. PubMed, Web of Science, ScienceDirect, and Google Scholar were the primary databases used to gather information from January 2023 to June 2025. The keywords or phrases used to search the information are “Haor livestock”, “Haor ecology”, “Climate change in Haor”, “Duck production in Haor*”*, “Livestock production in Haor”, “Livelihood of Haor people”, and so on. We have reviewed almost 150 articles, of which 50% have been included in this review paper.

### Expert consultation and validation

Valuable data and contextual insights were obtained through personal communication and consultations with field-level expert professionals. Specifically, livestock officers from the Department of Livestock Services (DLS) and other relevant livestock experts were consulted to obtain their thoughtful and insightful knowledge, ideas, and most up-to-date data. A total of 30 experts participated in the interview and discussion process.

### Collection, sorting, and presentation of temperature and rainfall data

Climatic data of temperature and rainfall were collected from the Climate Information Management System of the Bangladesh Agricultural Research Council [13]. Records spanning 72 years (1950–2022) were retrieved to identify long-term trends in temperature and rainfall in Sylhet district. The data were systematically sorted and organized by 5-year interval to identify the patterns in climatic behavior. Both the averages of temperature and rainfall were computed and graphically represented to illustrate the variations. The results are shown in the next Section.

### Case study: observation of crops and gizzard

A case study was conducted in two distinct Haor regions, Tarail in Kishoreganj and Dharmapasha in Sunamganj, to assess the overall nutritional status of ducks. A total of 36 ducks, with 18 from each location, were sampled at three specific times: 8:00 a.m., 12:00 p.m., and 4:00 p.m., across three age groups: 3, 6, and 12 months. To minimize the influence of sex, one male and one female duck were randomly selected for slaughter at each time point within each age group and location. Immediately after collection, the birds were weighed and then euthanized following the protocol described in the recently published paper by Afrin et al. [14]. The carcasses were opened, and the crops and gizzards were removed. Subsequently, the available feed ingredients within the contents were examined. The results are shown in the Section “Present Scenario of Livestock Production Potentials in Haor Regons”.

## Overall Climatic Dynamics and Their Impact on the Haor Ecosystem

As mentioned earlier, the Haor basin generally has a tropical monsoon climate characterized by heavy rainfall each year [1]. Among all the Haor districts, Sunamganj receives the most rainfall, being close to Cherrapunji, a place known for having the highest annual precipitation in the world, which measures 12 m. Data from 1960 to 2009 show that the Haor districts had a high average annual rainfall, with considerable variation across the region [15].

**Figure 2.**
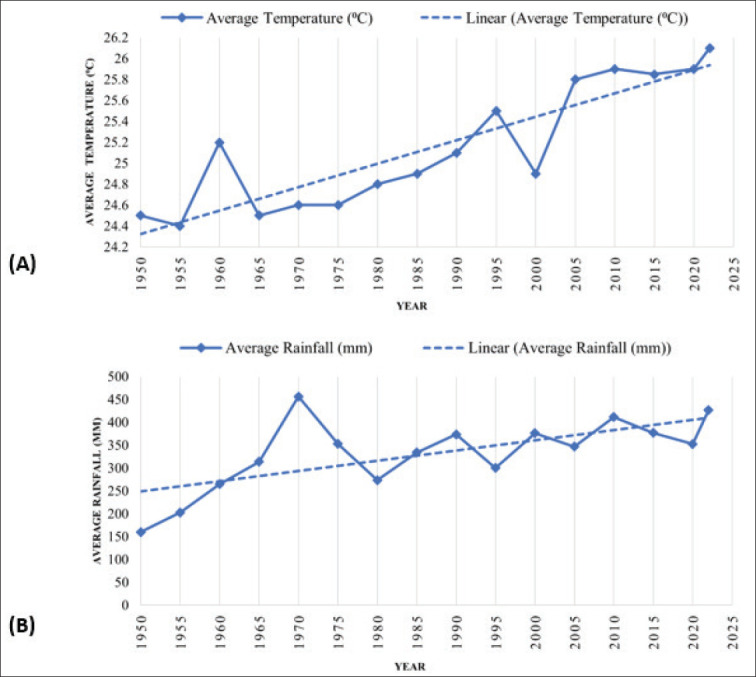
(A): Gradual increase of annual average temperature in Sylhet district for 72 years (1950–2022). (B): Gradual increase of annual average rainfall in Sylhet district for 72 years (1950–2022).

According to the Haor Master Plan 2 [15], the average monthly maximum temperature ranged from 25°C to 33°C, while the minimum monthly temperature ranged from 9°C to 26°C. Humidity levels fluctuate from 83% during the summer monsoon to 64% in the drier winter months [1]. The mean monthly evapotranspiration varies from 2.00 to 3.40 mm/day during dry seasons and from 3.90 to 4.80 mm/day during humid periods across the Haor region. From December to March, the prevailing wind comes from the northeast; from June to September, it shifts to the southwest. These two wind periods are known as the “northeast monsoon” and “southwest monsoon,” respectively [1]. April and May experience the highest daily maximum and average wind speeds, ranging from 26 to 37 km/h. We calculated the annual average temperature and rainfall in the Sylhet district of the Haor basin over a 72-year period (1950–2022). The raw data, obtained from the Climate Information Management System [13], were analyzed and presented in Figure 2A and B, for temperature and rainfall, respectively.

Results show an increase of 1.6°C in temperature and 266.5 mm in rainfall over the period. Several research studies have documented the overall impacts of climate change in Haor regions, noting a trend of rising temperatures and decreasing rainfall. Harris et al. [16] studied temperature and humidity changes in the Netrokona district over 38 years (1981–2018), finding a consistent but notable rise in temperature, especially after 2000, alongside a decreasing trend in precipitation.

It is worth noting that the data synthesized and analyzed from the Climate Information Management System [13] revealed a nearly stable or declining trend in precipitation across various regions of the country (Precipitation trends for the Dhaka and Rajshahi regions are provided as supplementary data (Supplementary Table 1). In contrast, the two- to threefold increase in rainfall recorded in Haor regions (Fig. 2B) may be a result of climate change in this particular region, which can be linked to the abrupt and unforeseen flash floods experienced in these areas over the past few years.

These climate changes undoubtedly have an adverse effect on crop and livestock production, aquaculture yields, forest ecosystems, and biodiversity. In recent years, the occurrence, patterns, and intensity of flash floods have undergone significant changes, posing a threat to the livelihoods of the Haor community. The degradation of the local environment and global climate change can be partially linked to the alteration of the Haor ecosystem, which in turn may contribute to the occurrence of diverse natural disasters. Several published reports also indicate the significant detrimental effects of climate change on agricultural production, livelihoods, and the well-being of people in Haor regions [17,18]. Recently published data also demonstrate that some farmers have successfully implemented agroecological tools, which have the potential to drive the transition in livestock production in the wetlands of Argentina [19]. Thus, considering the overall Haor ecosystem, a comprehensive policy intervention may be necessary to incorporate climate resilience strategies into crop, livestock, and fisheries production, as well as to adapt the local people to climate-related vulnerabilities.

## Institutional Contribution, Documentation, and Policy Adaptation for the Livelihood Improvement of Haor Peoples

The “Bangladesh Haor Development Board” was the first institution established by the government in 1977, which was later renamed as the Bangladesh Water and Wetland Development Board in 2000 through a resolution. It was decided to form this board as an attached department of the Ministry of Water Resources. After completing all formalities, the Ministry of Water Resources issued a notification on July 24th, 2016, to establish the Bangladesh Water and Wetland Development Directorate [20]. The Haor Master Plan, formulated by the Government of the People's Republic of Bangladesh in 2012, may currently be the only document under the board that specifically addresses critical issues such as wave erosion, flash floods, expansion of eco-friendly settlements, provision of housing for the poor, and rural livelihoods development for the Haor communities. The objective of the Haor Master Plan is to achieve sustainable development in the Haor region through integrated planning and the implementation of advanced climate resilience technologies, involving multiple stakeholders to protect vulnerable villages facing flash floods each year.

Additionally, the Haor and Char Development Institute (HCDI) was established at Bangladesh Agricultural University (BAU), Mymensingh 2202, Bangladesh. The mission of the HCDI is to develop high standards of agricultural professionals and provide needful assistance to increase agricultural production in Haor and Char areas through technological innovations and resource-oriented, demand-driven research activities. This institute currently offers a Master of Science in Wetland Agriculture degree, engaging in innovative research that addresses key challenges of Haor, with a focus on crops, fisheries, and livestock. Furthermore, the Department of Agronomy and Haor Agriculture at Sylhet Agricultural University, as well as the Department of Haor and Hill Agriculture at Habiganj Agricultural University, have also focused their research and academic efforts on various aspects of Haor agriculture. All these initiatives, such as the establishment of the government Haor Development Board, academic and research institutions at various public universities, and the significant documentation on Haor, have been accomplished within a short timeframe, collectively demonstrating the government's focused attention on the importance of the Haor economy. Now, we propose forming a high-level “Working Group” comprising prominent scientists, dynamic leaders, and relevant representatives from these academic, research, and government entities to exchange ideas and formulate and implement a comprehensive project necessary for the overall agricultural development in the Haor areas.

## Challenges in the Overall Livelihood of Haor People and Their Mitigation Practices

Over the past decade, the Haor region is believed to have contributed, on average, 2% of the country's GDP, with an average value of BDT 37,740 million, primarily from the agricultural sector [15]. The Haor basin has a relatively low population density compared to the rest of the country, with an average household size of 6.5, a birth rate of 3.2, and 35% of the population under the age of 10 [2]. The complex physical and hydrological features of the Haorhave kept this area still underdeveloped, despite being one of the key economic zones in the country. Farmers’ livelihoods in the Haor are considerably challenging and differ from those in other parts of the country in terms of ecology, financial activities, and overall agricultural production dynamics [6], primarily due to river erosion, poor road infrastructure, and sudden flash floods.

The cropping intensity in the Haor is around 147%, which is significantly lower than the national average of 182%. According to a recent report, the percentage of single-cropped areas (44.3%) in the Haor region was significantly higher than the national average of 26.1%. Regrettably, the proportion of single-cropped areas increased from 24% in 2007 to 44% in 2024, as calculated across all the Haor districts [8], indicating that the scope of diversified crop production has gradually decreased in the Haor regions. Further, the recent occurrence of sudden flash floods has caused significant damage to various agricultural and vegetable crops. This has resulted in delays in seedling growth, crop planting, and ultimately, the harvesting time, which have collectively had a negative impact on cropping intensity in Haor areas.

The Haor basin in Bangladesh is rich in fishery resources and serves as a vital habitat for many resident and migratory fish species, including 143 native and 12 exotic species, as well as various freshwater prawns [21]. Despite abundant natural resources and aquatic feed reserves, aquaculture production declined in the fiscal year 2021–2022 [22]. Fish populations face numerous threats, including frequent natural disasters, overfishing, overreliance on natural resources, low incomes for fishermen, political pressure, and inadequate enforcement of policies [21]. To mitigate the challenges mentioned above and improve the livelihoods of Haor people, several crucial issues must be addressed, including the adoption of modern technology, improved management practices, stopping the manipulation of river flows by narrowing rivers, preventing illegal sand mining, and carefully planning highway and culvert construction.

## Present Scenario of Livestock Production Potentials in Haor Regions

Table 1 illustrates the overall livestock population in the Haor districts of Bangladesh. As previously stated, Haor regions are submerged for more than 6 months each year, making them an ideal habitat for producing wetland birds, such as ducks and geese. Although livestock resources in the Haor regions include cattle, buffalo, goats, sheep, and chickens, the nomadic ducks and geese are relatively predominant in this region and significantly contribute to the livestock population, as both species require ample water for their growth, development, and reproduction. In contrast to chicken meat or egg production, these nomadic species are entirely dependent on natural aquatic feed resources, including snails, fish, pests, and aquatic weeds. The consumption of these natural feed materials by ducks and geese results in relatively safer duck meat, eggs, or goose meat for the nation. These products can be a choice for many consumers in the coming days, as there is considerable confusion regarding the quality of commercial broiler meat and other fast-growing chickens [23].

**Table 1.** Livestock population in the Haor districts of Bangladesh (in million).

District	Year	Cattle	Buffalo	Goat	Sheep	Chicken	Duck
Sunamganj	2016–17	0.36	0.02	0.12	0.14	3.45	1.30
	2023–24	0.37	0.02	0.12	0.16	4.08	1.65
	2024–25	0.38	0.02	0.12	0.16	4.17	1.70
Habiganj	2016–17	0.44	0.00	0.13	0.04	3.39	0.80
	2023–24	0.46	0.00	0.13	0.04	3.99	1.02
	2024–25	0.46	0.00	0.13	0.04	4.08	1.05
Moulvibazar	2016–17	0.25	0.02	0.18	0.03	2.43	0.50
	2023–24	0.26	0.02	0.19	0.03	2.89	0.63
	2024–25	0.26	0.02	0.19	0.03	2.95	0.65
Sylhet	2016–17	0.48	0.06	0.22	0.06	3.96	0.69
	2023–24	0.50	0.06	0.23	0.06	8.28	0.87
	2024–25	0.50	0.07	0.23	0.06	8.90	0.90
Kishoreganj	2016–17	0.32	0.01	0.09	0.03	4.86	0.91
	2023–24	0.33	0.01	0.10	0.03	5.71	1.16
	2024–25	0.33	0.01	0.10	0.03	5.83	1.19
Netrokona	2016–17	0.47	0.00	0.13	0.04	4.77	1.06
	2023–24	0.49	0.00	0.14	0.04	5.60	1.35
	2024–25	0.50	0.00	0.14	0.04	5.72	1.39
Brahmanbaria	2016–17	0.31	0.02	0.11	0.07	4.67	1.32
	2023–24	0.32	0.02	0.12	0.08	5.51	1.68
	2024–25	0.32	0.02	0.12	0.08	5.63	1.73

Source: DLS [28].

Homestead-based, in-house small- or medium-scale duck farming is widely practiced in Haor villages, primarily for meat and egg production. In a recent report, Islam et al. [24] mentioned the diverse approach of duck rearing in the Haor region, demonstrating that the majority of the farmers (41.11%) practiced a scavenging system, followed by semi-intensive (38.89%), lending (11.11%), and hardening methods (8.89%). The Haor districts are currently producing approximately 989 million units of eggs [25]. Several studies have shown that females in the family are often interested in poultry farming, and they may also engage in duck and animal rearing [26]. There are about 70.58 million ducks in Bangladesh, of which a significant number are reared in the Haor region [27,28]. Duck production in Haor areas, characterized by seasonal flooding and distinct ecological conditions, encounters numerous substantial challenges that greatly impact its sustainability and profitability. First, the low hatchability of duck eggs represents a significant concern, as evidenced by consistent observations in several research studies conducted in these regions [29]. This may result from multiple factors, including poor egg quality, inadequate parental management, traditional rice-husk incubation, and nutritional deficiencies within the breeding population. The hatchability of duck eggs in Haor regions was observed to be 65%–67% [29], which was much lower than the average hatchability of 87% recorded in the northern part of Bangladesh [30]. Second, duck mortality in Haor regions was found to be significantly higher (30%) compared to the plain land of Bangladesh (14.42%), as reported by Khan et al. [31] and Mostari et al. [32].

**Figure 3.**
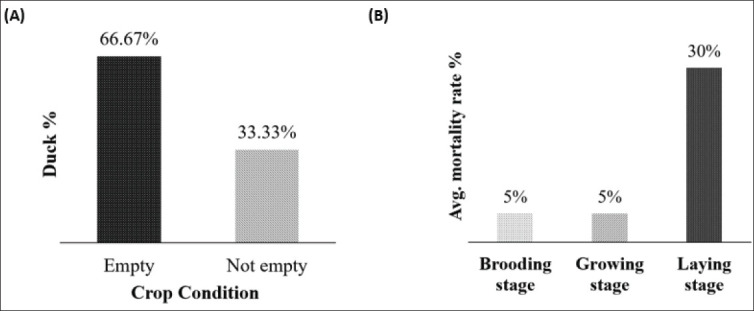
(A): Crop condition of the duck. (B): Duck mortality rate in the Haor.

The challenges of low hatchability and increased mortality have adversely affected the overall productivity and profitability of duck farming in the Haor districts. The principal factor contributing to these concerns is often associated with insufficient nutrition, inadequate disease management, and other pertinent management practices [33]. Limited access to diverse feed resources during specific seasons, particularly during flooding, may lead to nutrient deficiencies, resulting in increased disease susceptibility, higher mortality rates, reduced growth rates, and suboptimal productive and reproductive performances in ducks. To assess the nutritional status, we conducted a case study and collected crop and gizzard samples from 36 ducks at various time points and age groups from two separate locations in the Haor regions. We subsequently analyzed the availability of feed ingredients (methodology detailed in the Section “Materials and Methods”). Our data indicated that the majority of the crops and gizzards of the experimental ducks were empty (Fig. 3A). Further, our research group performed a comprehensive survey of small-scale duck farmers (*n* = 70) in several Haor regions (Kishoreganj, Sunamganj, and Netrokona districts) to evaluate the nutritional health of the ducks, farm management practices, and overall production potential. Notably, a significantly higher mortality rate of ducks was reported during the laying stage (Fig. 3B), which we hypothesize may be attributed to the severe suffering of the birds due to malnutrition. Furthermore, in-depth research is necessary to evaluate the impact of malnutrition on the productive and reproductive performance of ducks. Presumably, supplementation of adequate nutrition, especially during critical growth and reproductive phases, can significantly decrease mortality and enhance overall flock health, thereby potentially increasing egg production [34].

There are approximately 464.63 million heads of livestock in the country, of which around 50.25 million are found in the Haor region, which constitutes approximately 10.82% of the total livestock population [28]. Table 2 illustrates the meat, milk, and egg production trend in Haor regions of the country. Cattle are typically fed on rice straw, tree leaves, and rice bran. Food scarcity frequently occurs during the rainy season, resulting in the use of low-cost materials, such as water hyacinth and banana leaves, for animal feed, which can occasionally cause diarrhea. The fallow land between the paddy field and the wetland (*beel*) has traditionally served as pasture for the local cattle during the winter.

**Table 2.** Meat, milk (figure in MMT*), and egg (figure in million) production trend in the Haor region.

District		Year									
		2014–15	2015–16	2016–17	2017–18	2018–19	2019–20	2020–21	2021–22	2022–23	2023–24
Sunamganj	Meat	0.08	0.08	0.09	0.09	0.10	0.12	0.13	0.08	0.08	0.09
	Milk	0.06	0.09	0.10	0.10	0.10	0.13	0.18	0.06	0.09	0.10
	Egg	151.00	195.00	214.50	230.00	284.00	358.00	377.70	151.00	195.00	214.50
Habiganj	Meat	0.07	0.07	0.07	0.08	0.09	0.15	0.13	0.07	0.07	0.07
	Milk	0.07	0.07	0.08	0.09	0.09	0.14	0.18	0.07	0.07	0.08
	Egg	135.00	145.00	152.00	161.90	189.20	328.40	347.50	135.00	145.00	152.00
Netrokona	Meat	0.13	0.14	0.15	0.14	0.15	0.15	0.17	0.13	0.14	0.15
	Milk	0.16	0.17	0.15	0.15	0.16	0.24	0.29	0.16	0.17	0.15
	Egg	281.20	299.60	315.60	348.20	474.20	512.00	538.10	281.20	299.60	315.60
Moulvibazar	Meat	0.07	0.07	0.07	0.08	0.08	0.10	0.11	0.07	0.07	0.07
	Milk	0.07	0.07	0.07	0.08	0.08	0.13	0.16	0.07	0.07	0.07
	Egg	165.00	172.50	175.00	180.00	205.00	238.90	271.90	165.00	172.50	175.00
Brahmanbaria	Meat	0.07	0.09	0.09	0.08	0.13	0.13	0.16	0.07	0.09	0.09
	Milk	0.17	0.18	0.14	0.10	0.33	0.37	0.40	0.17	0.18	0.14
	Egg	107.50	96.60	124.20	134.50	231.30	283.10	279.80	107.50	96.60	124.20
Sylhet	Meat	0.04	0.06	0.07	0.09	0.12	0.13	0.16	0.04	0.06	0.07
	Milk	0.04	0.05	0.06	0.08	0.10	0.12	0.23	0.04	0.05	0.06
	Egg	85.00	105.00	121.50	167.60	258.00	335.00	449.20	85.00	105.00	121.50

MMT* = Million Metric Ton.

Source: DLS [28]

The Haor regions of Sylhet constitute one of the largest belts in Bangladesh, where buffalo husbandry is predominant among local farmers. Two primary types of buffalo are available in the country: riverine buffaloes, predominantly found in the southern coastal regions, and indigenous swamp buffaloes, which are prevalent in hilly and floodplain areas, such as Sylhet and Sunamganj in the northeast [35–37]. Published reports indicate that buffalo have ample access to naturally grown grasses during the dry season, when farmers do not provide supplementary feed, even during pregnancy and lactation. However, approximately 46.7% of additional concentrate is supplied to dairy buffalo during pregnancy and lactation in the rainy season [35]. The farmer's lack of knowledge regarding vaccinations, deworming, and other medications for the buffaloes is quite common in the Haor areas. The Black Bengal Goat (BBG) is a well-recognized goat breed in Bangladesh, primarily reared by rural households. It is esteemed for its compliance, early maturation, great prolificacy, and exceptional disease resistance, serving exclusively for meat production. The premier quality of BBG meat has been valued by local consumers for decades and is likely to remain unchanged. The market price of BBG meat is the highest among all animal protein meat sources. The BBG is also raised by rural households in the Haors, serving as a crucial source of income and sustenance for rural populations, providing meat and milk, frequently in conjunction with sheep in mixed herds.

Garole, a highly prolific sheep breed from India, possesses exceptional genetic potential for prolificacy, resistance to foot rot disease, and adaptation to grazing in knee-deep water, as well as hot, humid climates [38]. The breed was introduced to Bangladesh several years ago and is increasingly popular in different places, especially the Haor areas, where it is commonly raised by numerous small-scale farmers for profitable farming [39]. Table 3 illustrates the improved breeds/varieties of livestock and poultry, contemporary technology and management methods, vaccination, and disease control instruments employed by small-scale livestock farmers in the Haor areas.

**Table 3.** Improved breeds/varieties of livestock and poultry, modern technologies and management practices, vaccination, and disease control tools used by the small-scale livestock farmers in the Haor districts.

Sl. No.	List of improved breeds/varieties, and technologies	Purpose/benefits of using the improved technologies	References	Comments
1	Rearing of high-yielding exotic duck breeds (Jinding, Khaki Campbell, and Beijing).	Small and medium-scale farmers successfully raised modern exotic duck breeds within a scavenging system for egg and meat production. They also maintain duck parents for the production of hatching eggs.	Ali et al. [39]	Some farmers also raise indigenous black and white types of ducks for dual purpose.
2	The use of Baby Chick Ranikhet Disease Vaccine (BCRDV), Ranikhet Disease Vaccine (RDV), Duck Plague, Duck Cholera, and FMD vaccines.	These vaccines are used in the Haor areas to control specific diseases in designated avian or animal species. Farmers use these vaccines to prevent specific diseases, protect the herd/flock against further contamination, and reduce mortality, enabling them to secure a sustainable income over their investment.	Ali et al. [39]	The study was performed in the Sadar Upazila of Sunamganj district.
3	Preservation of feed and fodder throughout the arid season by the storage of grass, hay, and grain. Farmers additionally acquire feed for livestock during periods of feed scarcity.	Farmers preserve grass, hay, and corn to support livestock during floods and storms. It serves as an essential alternative when natural forage has been damaged by severe flash floods. The process of feed storage mitigates the risk of animal loss and saves farmers from the expense of emergency fodder purchases.	Fahim and Shikder [60]	In the Netrokona district of Haor, farmers with limited or no formal education are less willing to accept and apply scientific adaptation methods, preferring traditional approaches over those employed by their more educated counterparts.
4	Farmers’ engagement in beef fattening initiatives and Garole (sheep) rearing as key livestock operations.	In the Sadar Upazila of Sunamganj district, beef fattening improves cattle growth for greater meat production, while the rearing of Garole sheep increases farmers’ income by offering higher-quality meat and wool compared to traditional sheep.	Ali et al. [39]	Despite the challenges presented by recurrent floods, animal health and growth are progressively increasing due to nutrient supply, improved management, adequate vaccination, and deworming.
5	Urea Molasses Straw (UMS) supplementation to the bulls.	To increase the productivity of livestock and generate a profit. This feeding technique significantly improves growth performance in cattle by enhancing the nutritional value of cost-effective roughages, enabling farmers to achieve greater weight gain in shorter fattening periods.	Siddique et al. [61]	After the on-site experiment at Haor areas, the researchers have recommended UMS for better growth in a shorter fattening duration.
6	The expansion of the poultry industry, with numerous smallholders actively participating in commercial poultry farming in the Sunamganj district.	Implementing biosecurity measures helps mitigate disease outbreaks, reduces bird mortality, and enhances overall flock health and productivity. It increases egg yield, diminishes veterinary costs, and enhances agricultural profitability. Sanitary and secure measures enhance consumer trust and enable sustainable poultry farming.	Sani et al. [62]	The study indicated that the technology adoption by farmers was categorized into three groups: high adopters (17.78%, 8/45), partial adopters (57.76%, 26/45), and low adopters (24.44%, 11/45).
7	The use of traditional rice husk incubators for hatching duck eggs.	An established but efficient traditional method for incubating duck eggs in the Haor regions. It facilitates the attainment of satisfactory hatchability while meeting the rising demand for day-old ducklings. It is easily accessible to farmers and necessitates far lower operational costs compared to electric incubators, making it a cost-effective solution for sustainable duckling production.	Makaremuzzaman et al. [63]	It has the potential to develop into a significant Income Generating Asset (IGA) for numerous marginal farmers in the Haor region, provided adequate funding and appropriate guidance are available. Farmers even use this method to hatch duck eggs, despite the availability of electricity.
8	Establishment of the proposed ‘Cooperative Livestock Shelter’ in the Haor region.	It ensures the safety and welfare of animals during floods, safeguarding farmers’ livelihoods and supporting the long-term resilience of Haor communities. Until and unless a government or non-government initiative is taken, the farmers from neighbouring villages may establish a cooperative to manage animals in the proposed shelters.	Al Bayazid et al. [12]	The proposed ‘Cooperative Livestock Shelter’ primarily seems sound; however, there would be significant challenges in the practical implementation of such a complex concept.

## Research Advancement on Livestock Production, Disease Prevention Practices, and Control Measures

Despite significant advancements in crop, animal, and fish production in the Haor regions, as outlined in the Haor Master Plan 1 [27], there has been little attention paid to the overall research scope in this region. Although some scattered and segmented project activities have already been implemented in Haor regions aimed at boosting agricultural productivity, technological transfer, farming practices, and product marketing appropriateness, these initiatives may not produce tangible improvements in the livelihoods of Haor communities. The absence of cooperation in project design and alignment of primary objectives, insufficient monitoring and evaluation, and the lack of post-implementation assessment of societal implications are significant issues that must be addressed in agricultural research in Haors. Thus, before initiating any project in the Haor region, primary concerns are the consideration of the local ecosystem, conducting a comprehensive environmental impact assessment in accordance with global environmental and climate changes, engaging stakeholders for essential input, incorporating the green research design principles, biodiversity conservation, and finally, appropriate waste management strategies. Nonetheless, it remains a significant question whether the issues mentioned above were considered prior to the design of the projects that have already been implemented. Second, the relevant government authority should take the initiative to conduct a post-implementation impact analysis of major livestock development projects that have already been implemented, assessing their effectiveness, efficiency, and sustainability. This review paper has sorted out some relevant research articles published in national and international journals, categorized into “livestock production” and “treatment sections”, as illustrated in Tables 4 and 5, respectively.

**Table 4.** List of articles with major findings on livestock and poultry production in haor areas of Bangladesh.

Major findings	References
The authors stated that the major challenges for sustainable crop, livestock, and fish production in the Haor region are the low-lying lands, recurrent floods and flash floods, prolonged wetness, declining soil fertility, and the misuse of natural resources by local inhabitants.	Bokhtiar et al. [8]
The research encompassed 320 farmers in the Haor region of Bangladesh to examine their perspectives regarding climate change and adaptation techniques. The results unequivocally indicated that farmers have misconceptions regarding climate change, despite their perceptions being consistent with the facts of gradual and historical climatic changes.	Fahim and Sikder [60]
This study sought to assess the role of livestock in enhancing food security and alleviating poverty in the Haor regions of Bangladesh. The authors indicated that the livestock industry produced the highest annual household income in the Haor region.	Rahman et al. [64]
Despite the various unpredictable environmental conditions, such as flash floods posing considerable challenges every year, the relevant authorities may implement advanced livestock technologies compatible with the existing haor ecosystem.	Ali et al. [39]
The project entitled “Livelihood Improvement of Farming Community in Haor Area through System Approach,” implemented by BAU from 2010 to 2013, clearly stated that the integrated farming approaches significantly improved productivity, biodiversity, and farmers’ expertise in modern agricultural practices.	Karim and Bhuiya [65]
The study examined women’s participation in livestock and poultry management, focusing on essential income-generating activities in Hakaluki Haor, Bangladesh. Women demonstrated superior engagement compared to men, particularly in tasks related to feeding, managing, and marketing.	Mahadi et al. [66]
This study was conducted with Pekin, Muscovy, and Deshi White ducks to investigate the comparative performance of these three breeds under farmers’ management in the Haor region of Sylhet. Based on the results, the authors mentioned that Pekin ducks are preferable to Muscovy and Deshi White ducks.	Bhuiyan et al. [67]

**Table 5.** List of articles with major findings on livestock and poultry diseases in Haor areas of Bangladesh.

Major findings	References
About 60% of samples collected from the Haor regions of Habiganj and Brahmanbaria districts tested positive for the Duck Plague virus. The prevalence varied by organ, with the liver at 72%, the intestine at 64%, and oropharyngeal tissue at 44% in the birds.	Khan et al. [68]
The overall prevalence of GI parasites in the Sylhet division was 65.5%. The highest prevalence was found in Sylhet (78%), followed by Sunamganj (68%), Moulvibazar (66%), and Habiganj (50%) districts.	Ara et al. [50]
This paper outlines the prevalence, distribution, and risk factors associated with FMD in grazing cattle within Haor regions. The overall prevalence of FMD in grazing cattle was 24.71%, with a temporal pattern revealing a peak of 47.01% in June of that year.	Chowdhury et al. [49]
The study aimed to figure out the frequency of duck plague virus (DPV) in the haor regions. One hundred fifty-five cloacal swabs were collected from ducks in Netrokona, Kishoreganj, Brahmanbaria, and Sunamganj, with 41 samples (26.45%) confirming positive for DPV, demonstrating the highest frequency in Netrokona and Kishoreganj.	Hossen et al. [69]
A study of domestic ducks in Mymensingh and Sylhet revealed influenza type A antibodies in 60.73% and 47.73% of samples, respectively, with 17.5% testing positive for H5. Real-time RT-PCR revealed six type A viruses, including H1N5, H2N5, and low-pathogenic H7N5 from the Eurasian bird lineage.	Sarker et al. [70]
The prevalence of Fascioliasis was higher in females (41.36%) than in males (13.85%). Significantly *(p* < 0.01) higher prevalence of Fascioliasis was recorded in the winter season (51.33%), followed by the rainy (24.24%) and summer season (18.10%).	Afroze et al. [71]

## Impacts of Climate Change on Livestock and Fodder Crop Production in Haor Areas

Despite being an entirely agro-based nation with a rapidly expanding livestock sector, fodder crop production in Bangladesh faces significant challenges due to limited pastureland, the impacts of climate change, and a lack of awareness. Although Napier, Jumboo, Cowpea, and Pakchong are popular fodder varieties in Bangladesh [40], their cultivation and consumption are mostly limited to plain areas, with little availability in the Haor region. During the monsoon, the entire Haor is fully flooded, causing a severe shortage of pastureland that can lead to feed and fodder shortages for animals and birds [41]. As a result, large animals are confined to farmers' homesteads, subsisting mainly on rice straw, household waste, small amounts of concentrate, and often water hyacinth. Animals also access natural green grasses, leaves, or forages that begin to grow naturally early in winter as water recedes from the Haor. Farmers are pleased with the abundant feed resources that have become available until the next monsoon. Since Bangladesh is vulnerable to climate change—especially in Haor areas—the production and availability of animal feeds and fodders are increasingly affected. Rising sea levels exacerbate flooding by slowing the flow of inland rivers through backwater effects and lowering floodplain surface elevations relative to both sea level and riverbeds [40]. In flood-affected Haor regions, people face severe food crises due to the loss of crops, fish, ducks, and other resources caused by unexpected flash floods [42]. Heavy rains in March and April 2017, combined with upstream mountain floods, devastated approximately 90% of the cereal crops, vegetables, fruits, livestock, and fisheries across most Haor districts [43]. Such flash floods can also damage fodder crops and naturally grown grasses. These floods have affected around 1 million people and caused damage valued at up to $ 450 million to rice fields [44]. Flash floods tend to occur in an episodic manner, such as in recent years, including 2017, 2019, or 2022, rather than on a regular basis [45]. Recent gradual changes in ecological parameters like temperature, precipitation, humidity, and wind flow in Haor areas (as shown in Fig. 2A, B) may have led to flash flooding, resulting in pastureland scarcity, reduced feed and fodder production, and fewer aquatic feed resources—collectively complicating livestock and poultry farming [46]. The feed shortage is exacerbated by the conversion of fallow land into cultivated land, unregulated grazing, limited pasture access, and recurrent flooding, all of which contribute to a severe livestock feed crisis [26]. A survey revealed that about 221 of 378 farmers shifted from crop cultivation to livestock farming, while 192 sold their cattle to adapt to adverse conditions [47]. Again, the issue might be somewhat linked to the feed crisis of animals and birds. In highly vulnerable flood areas, 64.9% of cattle are severely affected, 27.9% moderately affected, and 7.1% less affected. In moderately vulnerable areas, 4.2% are severely affected, 70.8% are moderately affected, and 22.2% are less affected [47]. The causes of frequent flash floods in Bangladesh’s Haor basin are unclear; ecologists hypothesize that alongside global ecological impacts, infrastructure projects may impede natural water flow, thereby increasing the risk of flooding [48]. As a result, farmers struggle to predict floods due to upstream water control systems, sedimentation, and changes in river flow [47]. Therefore, it is recommended that authorities thoroughly assess ecological impacts before initiating new projects in Haor regions.

## Challenges of Climate-Resilient Livestock Production in Haor Regions and Possible Mitigation Approaches

Due to the land's vulnerability to floods and regular soil erosion during the rainy season each year, it is challenging to develop effective and suitable strategic planning, management practices, and their execution to accommodate the existing livestock populations [15]. The overall health of livestock and poultry in this region has deteriorated due to infectious diseases, inadequate or absent vaccination, insufficient treatment facilities, and occasionally the occurrence of diarrhea in large animals resulting from excessive consumption of water hyacinth [41]. Several factors may hinder the establishment of commercial livestock and poultry farming and its expected growth in the Haor region. However, inadequate transportation and communication infrastructure, insufficient marketing policies, and the assurance of reasonable profitability for livestock products to rural farmers should warrant significant attention from policymakers.

Outbreaks of Foot and Mouth Disease (FMD), the prevalence of liver fluke (Fascioliasis), and other gastrointestinal parasitic infestations in large animals are quite common in the Haor throughout the year, although the intensity of infection varies significantly based on seasonal changes, animal types, and specific locations of the Haor [49,50]. Hassan et al. [51] identified a potential cause of duck infections in Haor regions, noting a significant presence of migratory waterfowl, especially in Hakaluki and Tanguar Haors, where duck populations exhibit high seroprevalence and viral RNA prevalence. In addressing the recent challenges of unpredictable flash floods caused by climatic alterations, Al Bayazid et al. [12] described a transition from “flood control” to comprehensive “flood management” as essential, proposing temporary “flood shelters” for large animals. These shelters safeguard livestock during floods, ensuring animal welfare and protecting farmers’ livelihoods, while also strengthening the long-term resilience of Haor communities. Moreover, the potential for livestock production in Haor is constrained by several other factors, including the low genetic quality of animals and birds, insufficient quality input supplies, inadequate facilities and physical infrastructure for livestock management, structural deficiencies such as an ineffective regulatory framework, a shortage of skilled personnel and capital, and the failure to implement advanced scientific and technological innovations [5]. The overall temperature and rainfall in Haor regions (data derived from Sylhet district, depicted in Fig. 2A, B) have increased markedly over the past seven decades, and it is likely that similar climatic alterations will continue in the foreseeable future. Therefore, the overall livestock production strategies, often dependent on temperature, humidity, or average rainfall, must be rescheduled in alignment with the local ecological alterations. Consequently, the strategies for continuous feed and fodder production, the prevention and management of livestock diseases, the application of advanced scientific technologies, and all other sustainable solutions for enhanced livestock production must be evaluated in the context of possible climate change.

## Formulation of Livestock Development Strategic Plan for the Improvement of Haor Livelihoods

The Haor livestock resources remain largely unexplored and require effective management to achieve tangible outcomes that have the potential to significantly enhance the livelihoods of the Haor community [52]. Livestock production is indeed an integral aspect of Haor agriculture, extending beyond the mere production of animal protein in this region [39]. Zannat et al*.* [53] emphasized the importance of livestock, highlighting its role in facilitating self-employment, generating cash revenue, supplying fuel for rural households, producing organic manure for crop cultivation, and providing animal protein for human sustenance. Among the livestock species, the duck has been considered one of the most promising birds commonly raised under scavenging systems, primarily in Haor, low-lying, and coastal regions of the country. Despite the existence of several Government Regional Duck Breeding Farms in Haor regions, small-scale private farmers primarily depend on their self-developed methodologies and approaches for brooding, growing, and rearing ducks. They utilize rice husk-based traditional incubators for hatching duck eggs, manual sexing (where applicable), and breeding of parent stocks. Although there are a few regional government duck farms operating in the Haor regions, they may struggle to establish effective connections and requisite collaboration with small-scale duck farmers to explore suitable strategic policies for the production, processing, value addition, and marketing of day-old ducklings, duck eggs, and meat. Historically, rice husk-based egg incubation has been a traditional method employed in rural areas, where artificial incubators are often inoperable without electricity. Surprisingly, this technology is still used by farmers even after the availability of electricity in the particular region; thus, we suggest a comprehensive investigation into the efficacy, economic assessment, and knowledge, attitude, and practice of farmers regarding the adoption of new technology, as well as any other unidentified factors that hinder farmers from leveraging the requisite technological benefits that are available for the small-scale farmers at government regional duck breeding farms.

In addition to ducks, there are several prevalent and promising exotic breeds of chickens, such as Fayoumi, Rhode Island Red (RIR), crossbred Sonali, nondescript desi, and naked neck. Among them, Fayoumi exhibits a slightly superior performance in terms of weight gain, egg production, and livability [39], which may be further enhanced through appropriate strategic breeding, feeding, management, and disease control policies tailored to the local ecological environment. The Egyptian breed “Fayoumi” was imported into Bangladesh long ago and is potentially utilized as a maternal parent for the popular crossbred “Sonali” chicken, as well as being raised by rural farmers as part of their family poultry. The breed purity of Fayoumi and RIR is complicated by ongoing inbreeding and/or crossbreeding with native or exotic foreign breeds [14,54]. Therefore, we propose that the DLS undertake essential and suitable planning to import purebred Fayoumi and RIR, conserve their genetic resources at the regional poultry farm in Haor regions, and ensure a steady supply of Fayoumi and RIR DOCs as pure-line parents to the selected rural farmers.

Geese farming also exhibits promising prospects in Haor regions, characterized by ample opportunities for open foraging systems, numerous water bodies for unrestricted bird movement, and a lower risk of disease outbreaks. These factors collectively contribute to the feasibility of implementing goose rearing with minimal financial input. In exchange, on the other hand, farmers are provided with a consistent additional stream of revenue to cover their daily household expenditures. To explore the production potential and popularity of geese, the DLS should initiate the establishment of a “national geese breeding farm” in the Haor region to ensure a year-round supply of poults (baby geese) to farmers, possibly at a subsidized price. This initiative would serve as a source of income for impoverished farmers while also providing consumers with premium-quality goose meat.

**Figure 4.**
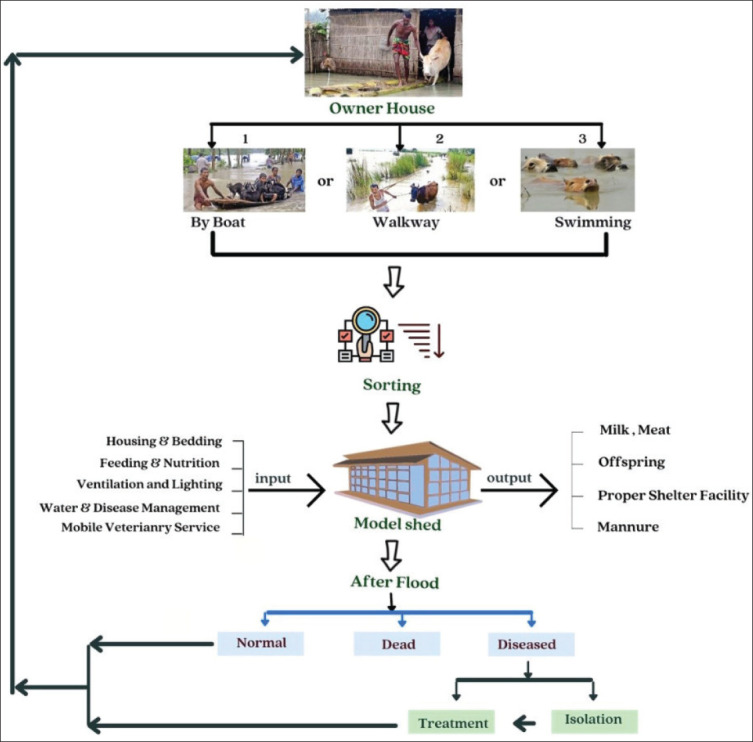
An operational flowchart of the proposed cooperative livestock shelter (Source: Al Bayazid et al. <a href=""><url>[12]</url></a>). This figure has been shown with permission from the corresponding author of the article.

The rearing of dairy cattle and buffaloes in the Haor region can be regarded as a lucrative aspect of livestock practices, due to the ample availability of government-owned fallow pasturelands. The rearing of large animals in Haor areas is critically crucial due to the acute scarcity of feed during the much longer waterlogging period each year. The potential of pastureland can be effectively utilized for producing low-cost, high-yielding green grasses during the dry season. Additionally, a portion of these grasses can be converted into silage, which may then be effectively used by farmers during periods of feed scarcity in the rainy season. Utilizing alternative treatment technologies, such as chaffing, soaking, or urea treatment, could enhance the nutritional content of paddy straw and preserve it for future use during times of feed scarcity. Taken together, all these efforts can transform traditional dairy cattle and buffalo production into a profitable and sustainable smart dairy enterprise [35]. Rearing goats and sheep have the potential to provide a substantial revenue stream for farmers, as these animals require minimal attention, maintenance, care, and investment [55]. Additionally, farmers residing in Haor areas can benefit from engaging in goat and sheep rearing activities. In a recent review article about the effects of floods on livestock farming in Haorregions, Al Bayazid et al*.* [12] highlighted the tremendous suffering experienced by livestock during the rainy season and suggested establishing a “cooperative livestock shelter” as a potential solution. The operational structure of the proposed cooperative livestock shelters, as outlined by the authors, is illustrated in Figure 4. We propose initiating at least one pilot program by the relevant government authorities to validate the feasibility of the “cooperative livestock shelters” concept, which aims to mitigate the critical problem of livestock rearing during the rainy seasons. Conceptually, as the authors proposed, farmers from two or three neighboring villages can establish a cooperative society to operate a shelter for their animals. The issue is time-demanding, although various obstacles are expected to arise during practical implementation.

Throughout history, several fermented milk products have been available to consumers in Bangladesh. Among these, Austagram cheese (also known as Dhaka cheese), originating from the Austagram upazila within the Kishoreganj district, has generated significant attention due to its distinctive taste, texture, flavor, and premium quality [56]. It is likely that the demand for homemade Austagram cheese, which is white-colored, salty, and soft, remains unchanged decade after decade. Due to its specific geographical origin, reputation, and unique characteristics, Austagram cheese has already been recognized as a “Geographical Indication” (GI) product [57]. Now, government initiatives may be employed to promote this unique dairy product countrywide and increase its popularity. Finally, contemporary need-based research, distribution of relevant technology, practical training for farmers, input support mechanisms, and effective marketing strategies for agricultural goods must be enhanced to elevate the productivity of all livestock and poultry [58]. Training may serve as an effective means of disseminating suitable livestock technologies to improve farmers' livelihoods [59].

The Haor Master Plan [15] was developed to promote sustainable development in Haor areas. It included several key components for the livestock sub-sector, such as enhancing the adaptive capacity of small and marginal farmers, encouraging community involvement in cattle and poultry farming, optimizing productivity, modernizing traditional livestock and livestock rearing methods, implementing integrated crop-livestock farming systems, establishing connections between smallholders and the market, adopting the one-house-one-farm model, and generating employment opportunities through livestock and poultry production and marketing. The Ministry of Fisheries and Livestock, the Department of Livestock Services, the Water Development Board, and all other relevant institutions should prioritize implementing the aforementioned planned activities. 

## Recommended Key Points Can be Considered for the Livestock Development in Haor Areas 

1. The livestock sector is also very lacking in contemporary technology, equipment, machinery for production, and information technology. Thus, we recommend implementing IoT-based advanced technologies, AI-integrated devices, and intelligent livestock tools, where suitable, to enhance the efficiency of livestock production in Haor regions.
2. The geomorphological characteristics and seasonal diversity caused the entire Haors region to be inundated for over 6 months annually, leading to numerous challenges in the marketing of agricultural products, such as fish, meat, milk, eggs, and various cereal grains. Livestock and fish products are mostly perishable. Thus, establishing market linkage is crucial for farmers to increase access and control over markets, as this has become a major challenge in protecting them from income erosion. To address this issue, a community-based cooperative society of local farmers might be established to ensure equitable pricing for agricultural products.
3. There are significant areas of Haor lands owned by the government (locally termed *Khas* land) that can be brought into green grass cultivation for livestock feeding. Again, government initiative is required to pilot grass production in a particular area through a cooperative society. Surplus grasses may be used for silage preparation, which would be supplied to the large animals during the acute feed scarcity in the rainy season.
4. The DLS should address the most potential but unexplored issues of duck farming through an appropriate and systematic duck breeding policy within small-scale duck farmers, including the pure breeds of commonly used exotic ducks (Jinding, Khaki Campbell, Pekin, and so on), proper nutritional management, vaccination, and disease control. An attempt should be made to import pure duck breeds and mass-vaccinate the ducks.
5. As described earlier, Al Bayazid et al. [12] propose establishing a ‘cooperative livestock shelter' in the Haor region to protect animals from flood susceptibility. In this context, despite potential challenges, a government or non-governmental organization may initiate a pilot program for the proposed cooperative shelter in a specific location to assess its feasibility.
6. Given the distinctive characteristics, reputation, and specific geographical origin of Austagramcheese, the Bangladeshi government should implement appropriate steps to promote this unique dairy product nationwide.
7. Wild birds, particularly migratory species in Haorregions, serve largely as reservoirs and vectors for diseases such as Avian Influenza and possible carriers of multi-drug-resistant bacteria. We suggest a comprehensive surveillance program to tackle the issues posed by these zoonotic diseases. Moreover, delineating and segregating the territorial zone for domestic ducks, geese, or other avian species from migrating wild birds can also be an effective strategy to mitigate the risk of avian influenza or other diseases.

## Conclusion

Regrettably, the Haor region of Bangladesh has historically lagged behind the overall national development, despite the rapid growth of Bangladesh's economy, infrastructure, and the general improvement in the livelihood of its mainstream people. The BDP 2100 has specifically identified the Haor region as one of six hotspots, which indeed unlocks the path for the protection and smart management of livestock and agricultural resources, ensuring the preservation of the natural environment and the well-being of the large population in the region. The underdeveloped livestock sector in the Haor region has the potential to enhance the country's economic growth and development through various means. These can be achieved through high-quality inputs, adequate services and infrastructure, access to low-interest loans, a strong regulatory framework and enforcement, proper training for skilled manpower, research focused on Haor issues, and technological advancements. Hence, it is imperative to formulate an independent and practical livestock development strategy for Haor, tailored to meet specific needs. This strategy should emphasize the integration and effective utilization of relevant resources while ensuring the preservation of local ecological balance, promoting economic well-being for communities, and enhancing resilience to climate change.

## Figures and Tables

**Supplementary Table 1.** Gradual increase of annual average rainfall in Rajshahi and Dhaka district for 72 years (1950–2022).

(a) Rajshahi																											
Parameters	Temperature (⁰C)														Rainfall (mm)												
Months Years	Jan.	Feb.	Mar.	Apr.	May	June	July	Aug.	Sept.	Oct.	Nov.	Dec.	Avg.	Jan.		Feb.	Mar.	Apr.	May	June	July	Aug.	Sept.	Oct.	Nov.	Dec.	Avg.
1965	18.9	20.8	24.6	28.3	29.7	29.5	28.8	28.8	29.0	27.1	23.8	19.1	**25.7**	0.0		21.0	61.0	22.0	80.0	325.0	404.0	227.0	171.0	75.0	19.0	0.0	**117.1**
1975	18.3	21.5	25.9	29.9	29.6	30.2	28.3	28.9	28.4	28.2	22.6	18.8	**25.9**	9.0		5.0	12.0	55.0	109.0	71.0	381.0	179.0	197.0	125.0	1.0	0.0	**95.3**
1985	18.5	19.9	27.2	30.6	28.2	29.6	28.3	29.2	28.9	27.0	22.7	19.5	**25.8**	4.0		7.0	3.0	71.0	190.0	232.0	274.0	146.0	233.0	77.0	3.0	12.0	**104.3**
1995	16.5	19.8	24.9	30.8	32.1	30.0	29.0	29.3	29.0	28.0	23.4	19.2	**26.0**	17.0		31.0	9.0	8.0	91.0	291.0	287.0	270.0	370.0	13.0	44.0	1.0	**119.3**
2005	17.6	22.0	26.3	29.6	29.7	31.1	29.2	30.0	29.9	26.5	22.4	19.7	**26.2**	14.0		1.0	104.0	27.0	108.0	92.0	492.0	161.0	131.0	275.0	0.0	0.0	**117.1**
2015	17.7	21.4	25.5	28.0	30.4	30.4	29.8	30.3	30.2	28.0	24.3	19.5	**26.3**	14.0		14.0	39.0	144.0	177.0	285.0	353.0	127.0	254.0	7.0	6.0	1.0	**118.4**
2022	17.8	19.3	27.3	31.2	29.5	30.6	31.7	30.7	30.1	28.4	23.7	20.2	**26.7**	3.0		39.0	0.0	20.0	222.0	70.0	60.0	263.0	329.0	57.0	0.0	1.0	**88.7**
(b) Dhaka																											
Parameters	Temperature (⁰C)														Rainfall (mm)												
Months Years	Jan.	Feb.	Mar.	Apr.	May	June	July	Aug.	Sept.	Oct.	Nov.	Dec.	Avg.	Jan.		Feb.	Mar.	Apr.	May	June	July	Aug.	Sept.	Oct.	Nov.	Dec.	Avg.
1965	18.8	21.1	25.2	28.8	29.5	28.2	28.2	27.8	28.4	27.1	23.6	19.9	**25.6**	0.0		28.0	22.0	55.0	305.0	442.0	304.0	480.0	300.0	50.0	131.0	0.0	**176.4**
1975	18.7	21.8	26.5	29.3	28.6	29.0	27.7	28.4	27.9	27.4	22.6	18.6	**25.5**	1.0		29.0	13.0	98.0	317.0	235.0	559.0	307.0	329.0	232.0	25.0	0.0	**178.8**
1985	20.4	22.6	28.4	29.1	28.2	28.9	28.3	29.2	28.8	28.2	24.6	21.7	**26.5**	8.0		1.0	195.0	176.0	300.0	399.0	262.0	317.0	306.0	79.0	0.0	10.0	**171.1**
1995	18.3	21.8	26.6	30.6	30.6	29.8	29.0	29.5	29.4	28.4	24.8	20.1	**26.6**	8.0		31.0	0.0	88.0	264.0	237.0	354.0	360.0	205.0	91.0	112.0	1.0	**145.9**
2005	19.4	23.7	27.3	29.3	28.7	30.1	28.6	29.4	29.4	27.5	24.4	21.4	**26.6**	1.0		3.0	155.0	91.0	291.0	259.0	542.0	361.0	514.0	417.0	3.0	0.0	**219.8**
2015	19.7	22.7	26.4	28.1	29.8	29.7	28.9	29.8	29.5	28.3	25.1	21.1	**26.6**	3.0		17.0	4.0	166.0	185.0	375.0	623.0	395.0	346.0	51.0	0.0	1.0	**180.5**
2022	20.2	21.6	28.5	30.0	29.5	30.2	31.0	30.8	29.9	28.8	25.9	22.3	**27.4**	12.0		20.0	14.0	106.0	217.0	168.0	51.0	130.0	282.0	297.0	0.0	4.0	**108.4**

## List of abbreviations

BARC, Bangladesh Agricultural Research Council; BAU, Bangladesh Agricultural University; BBG, Black Bengal goat; BBS, Bangladesh Bureau of Statistics; BDP, Bangladesh Delta Plan; BDT, Bangladeshi taka; BHWDB, Bangladesh Haor and Wetland Development Board; DOC, Day-old chicks; DoF, Department of Fisheries; DLS, Department of Livestock Services; FMD, Foot and mouth disease; GDP, Gross domestic product; HCDI, Haor and Char Development Institute; IFAD, International Fund for Agricultural Development; LSP, Livestock support providers; RIR, Rhode Island Red; RNA, Ribonucleic acid; USD, United States dollar; °C, degree Celsius; m, meter.

## References

[1] Kabir MH, Alim SMN (2007). Tanguar Haor: a diversified freshwater wetland.

[2] Sarma PK (2010). Scenario of Haor vulnerabilities and other obstacles for sustainable livelihood development in Nikli Upazila. J Bangladesh Agric Univ.

[3] Dey NC, Parvez M, Islam MR (2021). A study on the impact of the 2017 early monsoon flash flood: potential measures to safeguard livelihoods from extreme climate events in the Haor area of Bangladesh. Int J Disaster Risk Reduct.

[4] Salauddin M, Islam AK (2011). Identification of land cover changes of the Haor area of Bangladesh using Modis Images. In 3rd International Conference on Water u0026amp; Flood Management (ICWFM-2011).

[5] SKI Suvra KI (2021). Haor regions-importance, problems, strategy and future development. J Econ Finance.

[6] Alam MS, Quayum MA, Islam MA (2010). Crop production in the Haor areas of Bangladesh: insights from farm level survey. Agriculturists.

[7] Chakraborty D, Mondal KP, Islam ST, Roy J, Shrestha S, Djalante R, Shaw R, Pal I (2021). 2017 flash flood in Bangladesh: lessons learnt. Disaster resilience and sustainability.

[8] Bokhtiar SM, Islam MJ, Samsuzzaman S, Jahiruddin M, Panaullah GM, Salam MA, et al. (2024). Constraints and opportunities of agricultural development in Haor ecosystem of Bangladesh. Ecologies.

[9] Mitra C, Rahman KMM, Haque MR, Hashem MA (2020). Rearing and marketing of livestock in the Hakaluki Haor: impact on livelihood, food and nutrition security. J Agric Food Environ.

[10] Parvez M, Islam MR, Dey NC (2022). Household food insecurity after the early monsoon flash flood of 2017 among wetland (Haor) communities of northeastern Bangladesh: a cross-sectional study. Food Energy Secur.

[11] IFAD (2021). Protecting homes and livelihoods in Bangladesh’s Haor basin.

[12] Al Bayazid A, Harun AB, Billah MM, Afrin M, Ali MZ, Meher MM (2025). Impact of flood on livestock farming and a possible solution to reduce the suffering of livestock in the Haor region of Bangladesh. J Res Vet Sci.

[13] BARC (2025). Climate information management system.

[14] Afrin A,  Ahmed T,  Lahiry A,  Rahman S,  Dey B,  Hashem MA, et al. (2024). Profitability and meat quality of fast-, medium- and slow-growing meat-type chicken genotypes as affected by growth and length of rearing. Saudi J Biol Sci.

[15] Bangladesh Haor and Wetland Development Board (2012). Haor master plan. https://dbhwd.portal.gov.bd/sites/default/files/files/dbhwd.portal.gov.bd/publications/298d5166_988c_4589_96cb_36e143deba4f/Haor%20Master%20Plan%20Volume%202.pdf.

[16] Harris IC, Jones PD, Osborn T (2017). Climatic Research Unit (CRU) time-series (TS) version 4.01 of high-resolution gridded data of month-by-month variation in climate (Jan. 1901–Dec. 2016). Centre for Environmental Data Analysis.

[17] Bari SH, Husna NEA (2022). People’s perception on climate change effects and adaptation in the haor basin of Bangladesh. Int J Hydrol Sci Technol.

[18] Shajahan KM, Bista RB (2023). Climate change effects on livelihood resilience of Haor people in Mohanganj Upazila. J Entrep Bus Resil.

[19] Muchiutti AA, Jacobo E, Quintana R, Attademo AM (2025). Livestock production systems in wetlands of Argentina: assessing transition toward sustainable agroecological systems. Agroecol Sustain Food Syst.

[20] DBHWD (2025). About us. Department of Bangladesh Haor and Wetland Development Board, Dhaka, Bangladesh.

[21] AR Sunny, Masum KM, Islam N, Rahman M, Rahman A, Islam J, et al. (2020). Analyzing livelihood sustainability of climate vulnerable fishers: insight from Bangladesh. J Aquacult Res Dev.

[22] DoF (2022). Yearbook of fisheries statistics of Bangladesh, 2021-2022. Fisheries Resources Survey System (FRSS), Ministry of Fisheries and Livestock.

[23] Das SC, Tasmin MZ, Afrin A, Ahmed T, Lahiry A, Rahman S (2024). Challenges in the profitability of small-scale broiler farming by avoiding injudicious use of drugs and additives. Heliyon.

[24] Islam S, Sultana S, Islam MA, Sarker MSK, R. Khatun (2025). Existing scenario and profitability in duck farming in selected Haor and coastal areas of Bangladesh. Am J Aquacult Anim Sci.

[25] Seastedt TR (2025). Accelerating contributions of restoration ecology for enhancing natural climate solutions. Acad Biol.

[26] Rabby TG, Fredericks LJ, Alam GM (2013). Livestock husbandry strategy in alleviating poverty in the haor area of Bangladesh. Asian J Anim Vet Adv.

[27] Bangladesh Haor and Wetland Development Board (2023). Haor master plan.

[28] DLS Livestock economy at a glance (2024-2025). Department of Livestock Services, Ministry of Fisheries and Livestock, GoB, Dhaka, Bangladesh, 2025.

[29] Sheheli S, Mithun MN, Banik S (2023). Profitability and problems of farmers in duck farming: a study on haor areas in Bangladesh. Int J Agric Sci Res Technol Ext Educ Syst.

[30] Zahan MN, Sufian MK, Rahman MK, Parvej MS (2016). Socio-economic status of farmers and production performance of Khaki Campbell ducks reared under backyard farming in Bangladesh. Wayamba J Anim Sci.

[31] Khan K, Saha S, Hossain M, Haque M, Haq M, Islam M (2018). Epidemiological investigation of recurrent outbreaks of duck plague in selected Haor (wetland) areas of Bangladesh. J Adv Vet Anim Res.

[32] Mostari S, Rashid SH, Ali MH, Akther M, Islam MN (2021). Clinicopathological status of duck plague at Dinajpur district of Bangladesh. Asian Australas J Biosci Biotechnol.

[33] Abd El-hack ME, Hurtado CB, Toro DM, Alagawany M, Abdelfattah EM, Elnesr SS (2019). Fertility and hatchability in duck eggs. World Poult Sci J.

[34] Kabir F, Rahman A, Biswas H (2020). A study on production performance of local ducks and identifying the constraints of duck rearing at farmer’s level. Asian J Res Anim Vet Sci.

[35] Famous M, Aditya AC, Ahmed S, Sutradhar S (2021). Productive and reproductive performance of different crossbred dairy cattle at Kishoreganj, Bangladesh. Vet Sci Res Rev.

[36] Sarker A, Biswas D, Fakruzzaman M, Deb GK, Hossain SMJ, Alam MA, et al. (2024). Enhancement of the pregnancy rate of buffalo cows through intra-vaginal bio-stimulation with penis-like device in the coastal area of Bangladesh. Adv Anim Vet Sci.

[37] Pehan EA, Miah M, Rahman MH, Shejuty SF, Haque MN, Huda MN, et al. (2025). A holistic review of buffalo productivity, reproductive efficiency, genetic improvement, and disease management in Bangladesh. Vet Anim Sci.

[38] Roy I, Rahman M, Behera R, Karunakaran M, Mandal A (2023). Garole-a promising sheep breed in coastal West Bengal. J Indian Soc Coastal Agric Res.

[39] Ali S, Kashem MA, Aziz MA (2018). A scenario of agricultural technologies practiced in Haor area of Sunamganj district in Bangladesh. Seedling.

[40] Uddin MT, Reza M, Dhar AR (2023). Green grass production for improving farmers’ socioeconomic status in major milk pocket areas of Bangladesh. World Dev Sustain.

[41] Sadeque AZ (2019). Indigenous flood coping strategies towards livelihoods: a study in haor area of North-East Bangladesh. Doctoral dissertation, University of Dhaka.

[42] Tashmin N, Khan ATMJ (2018). Untimely visitation of calamity: flash flood in Haor areas of Bangladesh and its devastating impacts on economy. Soc Change.

[43] Department of Livestock Services (DLS) (2021). Term of reference (TOR) for consulting firm to conduct benchmark with baseline survey under Integrated Livestock Development Project in Haor areas.

[44] Kamal ASMM, Shamsudduha M, Ahmed B, Hassan SMK, Islam MS, Kelman I, et al. (2018). Resilience to flash floods in wetland communities of northeastern Bangladesh. Int J Disaster Risk Reduct.

[45] Akter N, Islam MR, Karima MA, Miah MG, Rahman MM (2023). Spatiotemporal rainfall variability and its relationship to flash flood risk in northeastern Sylhet haor of Bangladesh. J Water Clim Change.

[46] Thomas TS, Mainuddin K, Chiang C, Rahman A, Haque A, Islam N (2013). Agriculture and adaptation in Bangladesh: current and projected impacts of climate change. IFPRI Discussion Paper No. 01281.

[47] Akter N, Islam MR, Karim MA, Miah MG, Rahman MM (2022). Impact of flash floods on agri-based livelihoods in Sylhet haor basin. Ann Bangladesh Agric.

[48] Kamruzzaman M, Shaw R (2018). Flood and sustainable agriculture in the Haor basin of Bangladesh: a review paper. Universal J Agric Res.

[49] Chowdhury MS, Ahsan MI, Khan MJ, Rahman MM, Hossain MM, Harun-Al-Rashid A (2020). Data on prevalence, distribution and risk factors for foot and mouth disease in grazing cattle in Haor areas of Bangladesh. Data Brief.

[50] Ara I, Ahmed J, Dipta PM, Nath SD, Akter T, Adnan MR, et al. (2021). Prevalence and severity of gastro-intestinal parasites in buffalo calves at Sylhet division of Bangladesh. J Parasit Dis.

[51] Hassan MM, Islam A, Hasan RB, Rahman MK, Webby RJ, Hoque MA, et al. (2020). Prevalence and distribution of avian influenza viruses in domestic ducks at the waterfowl-chicken interface in wetlands. Pathogens.

[52] Uddin M, Hossain M, Rahman M, Sarker S (2020). Participation of farmers in resource management activities at selected Haor areas in Netrokona district. J Bangladesh Agric Univ.

[53] Zannat M, Sharmin S, Tama RAZ, Akteruzzaman M (2018). An economic study on production and marketing of ducks in Haor areas of Netrokona district. Res Agric Livest Fish.

[54] Das SC, Chowdhury SD, Khatun MA, Nishibori M, Isobe N, Yoshimura Y (2008). Poultry production profile and expected future projection in Bangladesh. World Poult Sci J.

[55] Mowsume SA, Khandoker MA, Ahmed S, Disha HN, Mahbubul M, Khatun A, et al. (2023). Growth performance of native sheep under semi-intensive production system in Bangladesh. J Livest Res.

[56] Sultana T, Adib SS, Al Emam M, Imran A, Oishee FF, Rashid MHU, et al. (2025). Characterization of artisanal Bangladeshi Austagram cheese: physicochemical, colour, textural, sensorial, and functional properties. Int Dairy J.

[57] Zamir M (2024). GI products and Bangladesh’s economic diplomacy. Financial Express.

[58] Chander MC, Dutt TDT, Ravikumar RK, Subrahmanyeswari B (2010). Livestock technology transfer service in India: a review. Indian J Anim Sci.

[59] Hossain M, Islam M, Akhter A, Rashiduzzaman M (2021). Impact of training on livestock technology transfer for rural poor farmers livelihood improvement in Bangladesh. SAARC J Agric.

[60] Fahim TC, Sikder BB (2022). Exploring farmers’ perception of climate-induced events and adaptation practices to protect crop production and livestock farming in the Haor area of north-eastern Bangladesh. Theor Appl Climatol.

[61] Siddique MA, Khan M, Roy S, Kashem MA (2018). Effect of urea molasses straw on beef cattle fattening and income generation of small-scale farmers in Haor areas. J Sylhet Agric Univ.

[62] Sani NS, Ahmed S, Akter S, Ahsan MI (2021). Adaptation level of biosecurity measures by layer smallholders in Sunamganj district, Bangladesh: a cross-sectional survey. J Sylhet Agric Univ.

[63] Makaremuzzaman M, Hasnath MR, Miah MY, Belal SA, Hasan MK (2016). Study on rice husk incubation for duckling production in Sunamganj district of Bangladesh. Int J Agric Biosci.

[64] Rahman KM, Hossain MJ, Rana MS (2020). Livestock and poultry rearing by smallholder farmers in Haor areas in Bangladesh: impact on food security and poverty alleviation. Bangladesh J Agricult Econ.

[65] Karim MM, Bhuiya MS (2017). Poverty alleviation of farming community in haor area through farming systems. Asian J Poverty Stud.

[66] Mahadi MS, Khanum R, Akhi K (2014). Participation in livestock and poultry rearing: a study on haor women in Bangladesh. J Chem Biol Phys Sci.

[67] Bhuiyan MM, Khan MH, Khan MA, Das BC, Lucky NS, Uddin MB (2005). A study on the comparative performance of different breeds of broiler ducks under farmer’s condition at farming system research and development (FSRD) site, Sylhet, Bangladesh. Int J Poult Sci.

[68] Khan KA, Islam MA, Sabuj AAM, Bashar MA, Islam MS, Hossain MG, et al. (2021). Molecular characterization of duck plague virus from selected Haor areas of Bangladesh. Open Vet J.

[69] Hossen A, Rahman M, Ali M, Yousuf M, Hassan M, Giasuddin M (2019). Investigation of duck plague virus in Hoar areas of Bangladesh. Bangladesh J Livest Res.

[70] Sarker RD, Giasuddin M, Chowdhury EH, Islam MR (2017). Serological and virological surveillance of avian influenza virus in domestic ducks of the north-east region of Bangladesh. BMC Vet Res.

[71] Affroze S, Begum N, Islam M, Rony S, Islam M, Mondal M, et al. (2013). Risk factors and gross pathology of bovine liver fluke infection at Netrokona district, Bangladesh. J Anim Sci Adv.

